# Notes on the Treatment of Charged Particles for Studying Cyclotide/Membrane Interactions with Dissipative Particle Dynamics

**DOI:** 10.3390/membranes12060619

**Published:** 2022-06-14

**Authors:** Felix Bänsch, Christoph Steinbeck, Achim Zielesny

**Affiliations:** 1 Institute for Bioinformatics and Chemoinformatics, Westphalian University of Applied Sciences, August-Schmidt-Ring 10, 45665 Recklinghausen, Germany; felix.baensch@w-hs.de; 2 Institute for Inorganic and Analytical Chemistry, Friedrich-Schiller-University Jena, Lessing Str. 8, 07743 Jena, Germany; christoph.steinbeck@uni-jena.de

**Keywords:** cyclotide, biological membrane, plasma membrane, membrane disruption, electrostatic interaction, charge, ion pairing, dissipative particle dynamics, DPD

## Abstract

Different charge treatment approaches are examined for cyclotide-induced plasma membrane disruption by lipid extraction studied with dissipative particle dynamics. A pure Coulomb approach with truncated forces tuned to avoid individual strong ion pairing still reveals hidden statistical pairing effects that may lead to artificial membrane stabilization or distortion of cyclotide activity depending on the cyclotide’s charge state. While qualitative behavior is not affected in an apparent manner, more sensitive quantitative evaluations can be systematically biased. The findings suggest a charge smearing of point charges by an adequate charge distribution. For large mesoscopic simulation boxes, approximations for the Ewald sum to account for mirror charges due to periodic boundary conditions are of negligible influence.

## 1. Introduction

Biomolecular membrane processes often take place on a microsecond scale, involving large interacting molecular ensembles with millions of atoms. Consequently, an atomic resolution modeling approach requires billions of integration time steps, with each time step accounting for all mutual atomic interactions based on approximate molecular mechanics force fields. Corresponding simulation runs need substantial computational resources, with simulation times often in the order of weeks or months [1]. In contrast, coarse-grained mesoscopic simulation techniques such as dissipative particle dynamics (DPD) considerably reduce the necessary number of interacting particles and allow much longer integration time steps on the picosecond scale, as soft particle–particle interactions replace their hard atomistic equivalents. As a result, mesoscopic simulations are orders of magnitude faster, with simulation runs of molecular ensembles representing millions of atoms for microseconds being completed within hours or days on standard multicore workstations [2,3]. Conversely, mesoscopic simulations imply a much lower resolution above the atomic level with only isotropic particle–particle interactions that average nonbonded interactions at the atomic scale—limitations that may prevent an adequate description of the molecular processes in question. Moreover, for an adequate representation of biomolecular membrane compounds or peptides and proteins, additional charged particles should be introduced, with long-range electrostatic interactions that superimpose the concurrent short-range interaction framework. However, hard electrostatic interactions between charged particles are alien to a soft interacting system and may lead to possible unphysical artifacts such as artificial ion pairing between differently charged particles [4].

This communication discusses the established approaches to integrating charged particles into a soft DPD context involving the interaction of cyclic peptides (cyclotides) with biological plasma membranes. Cyclotides exhibit collaborative membrane-disrupting activities on the microsecond scale [5]. Membrane lipid extraction as a specific mode of action [6,7,8,9] can be successfully investigated by DPD simulations [10], which, in particular, allow quantitative assessment of the dynamics of membrane disruption to characterize a specific cyclotide/membrane system for comparative purposes [11]. The agreement of quantitative lipid extraction within a cyclotide/membrane “sandwich” model (see details below) with experimental trends was demonstrated for more than two dozen cyclotide/membrane systems. Moreover, the linear additivity of cyclotide activity for cyclotide mixtures could be successfully modeled. In this work, the cyclotide/membrane “sandwich” model is utilized for an evaluation of the different approaches to charge treatment to promote mesoscopic modeling of biomolecular membrane processes.

## 2. Methods

Dissipative particle dynamics (DPD) is a mesoscopic simulation technique for isothermal complex fluids and soft matter systems. It satisfies Galilean invariance and isotropy, conserves mass and momentum, and achieves a rigorous sampling of the canonical NVT ensemble due to soft particle pair potentials that diminish molecular entanglements or caging effects. DPD is expected to show correct hydrodynamic behavior and to obey the Navier–Stokes equations [12,13,14,15,16]. DPD particle trajectories are guided by Newton’s equation of motion

$$m_{i}\frac{d^{2}{\underline{r}}_{i}}{dt^{2}}={\underline{F}}_{i}=\sum_{\begin{array}{l} j=1\\ j\neq i \end{array}}^{N}\left({\underline{F}}_{ij}^{DPD}+{\underline{F}}_{ij}^{D}+{\underline{F}}_{ij}^{R}+{\underline{F}}_{ij}^{B}+{\underline{F}}_{ij}^{E}\right)$$

$m_{i},{\underline{r}}_{i}$, mass and spatial position of particle *i*; *t*, time; ${\underline{F}}_{i}$, total force on particle *i* exerted by other particles *j*;
${\underline{F}}_{ij}^{DPD}$, conservative soft repulsive DPD force on particle *i* exerted by particle *j*; ${\underline{F}}_{ij}^{D}$, dissipative (frictional) force;
${\underline{F}}_{ij}^{R}$, random force; ${\underline{F}}_{ij}^{B}$, conservative harmonic bond force; ${\underline{F}}_{ij}^{E}$, conservative electrostatic force.

where the total force on a particle exerted by other particles consists of a conservative, a dissipative (frictional), and a random part. The opposing dissipative and random forces depend on each other and act as a thermostat conserving the total momentum and introducing Brownian motion into the system. The conservative forces comprise soft DPD particle repulsions that represent averaged nonbonding interactions of uncharged DPD particles as well as possible harmonic springs between bonded or electrostatic interactions between charged particles.

DPD particles, in general, may be arbitrarily defined as fluid packets [13]. Molecular fragment DPD is a bottom-up variant [16,17,18,19,20,21,22] that identifies each particle with a distinct small molecule of molar mass in the order of 100 Da. Larger molecules are composed of adequate smaller molecular fragment particles that are bonded by harmonic springs to mimic covalent connectivity and spatial 3D conformations. 

For charge treatment within the DPD framework [4,23,24,25,26], the electrostatic potential between two charged particles can be neatly arranged as a product of a classical Coulomb term (denoted with index C), a charge distribution term (index D), and a splitting term (index S):

$$U^{E}\left(r_{ij}\right)=t_{C}\left(r_{ij}\right)t_{D}\left(r_{ij}\right)t_{S}\left(r_{ij}\right)$$

$U^{E},\;\mathrm{electrostatic}\;\mathrm{potential}\;\mathrm{energy}\;\mathrm{in}\;\mathrm{reduced}\;\mathrm{DPD}\;\mathrm{units};$
$r_{ij},\;\mathrm{distance}\;\mathrm{between}\;\mathrm{particles}\;i\;\mathrm{and}\;j\;\mathrm{in}\;\mathrm{reduced}\;\mathrm{DPD}\;\mathrm{length}\;\mathrm{units}.$

The Coulomb term is given by

$$t_{C}\left(r_{ij}\right)=\Gamma \frac{Z_{i}Z_{j}}{r_{ij}}$$

$Z_{i},\;\mathrm{Valence}\;\mathrm{of}\;\mathrm{charged}\;\mathrm{particle}\;i.$

The dimensionless electrostatics coupling constant [4] evaluates to

$$\Gamma =\frac{e^{2}}{4\pi k_{B}T\varepsilon_{0}\varepsilon_{r}R_{cutoff}}\approx \frac{167100.946898}{\varepsilon_{r}\left(\frac{T}{K}\right)\frac{R_{cutoff}}{Å}\frac{T}{K}}$$

$e,\;\mathrm{elementary}\;\mathrm{charge};\;k_{B},\;\mathrm{Boltzmann}\;\mathrm{constant};\;T,\;\mathrm{temperature};\;\varepsilon_{0},\;\mathrm{vacuum}\;\mathrm{permittivity};$
$\varepsilon_{r},\;\mathrm{relative}\;\mathrm{permittivity};\;R_{cutoff},\;\mathrm{DPD}\;\mathrm{cut-}\mathrm{off}\;\mathrm{length}.$

The temperature dependence of the relative permittivity for water can be approximated by [27]

$$\varepsilon_{r}\left(\frac{T}{K}\right)\approx 295.87696+\left(\frac{T}{K}\right)\left(-1.229097+\left(\frac{T}{K}\right)\left(0.0020952245-0.00000141\left(\frac{T}{K}\right)\right)\right)$$

The charge distribution term turns the particle point charges into “smeared” charge distributions with a defined decay length [25]

$$t_{D}\left(r_{ij}\right)=1-\left(1+\frac{r_{ij}}{\lambda }\right)\exp\left\{-\frac{2r_{ij}}{\lambda }\right\}$$

λ, decay length of the charge (λ = Γ in reduced DPD length units).

The splitting term (SP3 splitting in [26]) approximates the effect of mirror charges due to periodic boundary conditions (otherwise considered by the Ewald sum [23,24,26]):

$$t_{S}\left(r_{ij}\right)=1-\frac{7}{4}\left(\frac{r_{ij}}{R_{cutoff,el}}\right)+\frac{21}{4}{\left(\frac{r_{ij}}{R_{cutoff,el}}\right)}^{5}-7{\left(\frac{r_{ij}}{R_{cutoff,el}}\right)}^{6}+\frac{5}{2}{\left(\frac{r_{ij}}{R_{cutoff,el}}\right)}^{7}$$

$R_{cutoff,el},\;\mathrm{electrostatics}\;\mathrm{cut-}\mathrm{off}\;\mathrm{radius}\;\mathrm{in}\;\mathrm{reduced}\;\mathrm{DPD}\;\mathrm{units}.$

The electrostatic forces on the charged particles are calculated by the derivatives of the electrostatic potential:

$$\begin{array}{l} {\underline{F}}_{ij}^{E}\left(r_{ij}\right)=-\frac{dU^{E}\left(r_{ij}\right)}{dr_{ij}}{\underline{r}}_{ij}^{0}\\ {\underline{F}}_{ij}^{E}\left(r_{ij}\right)=-\left(\frac{dt_{C}\left(r_{ij}\right)}{dr_{ij}}t_{D}\left(r_{ij}\right)t_{S}\left(r_{ij}\right)+t_{C}\left(r_{ij}\right)\frac{dt_{D}\left(r_{ij}\right)}{dr_{ij}}t_{S}\left(r_{ij}\right)+t_{C}\left(r_{ij}\right)t_{D}\left(r_{ij}\right)\frac{dt_{S}\left(r_{ij}\right)}{dr_{ij}}\right){\underline{r}}_{ij}^{0} \end{array}$$

${\underline{F}}_{ij}^{E},\;\mathrm{Electrostatic}\;\mathrm{force}\;\mathrm{on}\;\mathrm{particle}\;i\;\mathrm{exerted}\;\mathrm{by}\;\mathrm{particle}\;j\;\mathrm{in}\;\mathrm{reduced}\;\mathrm{DPD}\;\mathrm{units};$
${\underline{r}}_{ij}^{0},\;\mathrm{unit}\;\mathrm{vector}\;\mathrm{that}\;\mathrm{points}\;\mathrm{from}\;\mathrm{particle}\;j\;\mathrm{to}\;\mathrm{particle}\;i.$

If charge distribution and splitting are not taken into account, their corresponding terms are set to one (the pure Coulomb term remains, which will be denoted C); otherwise, the combinations of the Coulomb term with charge distribution (denoted CD) and splitting in addition (denoted CDS) are considered. Thus, the forces of the three different approaches evaluate to

$$\begin{array}{l} {\underline{F}}_{ij}^{E\left(C\right)}\left(r_{ij}\right)=-\frac{dt_{C}\left(r_{ij}\right)}{dr_{ij}}{\underline{r}}_{ij}^{0}\\ \frac{dt_{C}\left(r_{ij}\right)}{dr_{ij}}=-\Gamma \frac{Z_{i}Z_{j}}{r_{ij}^{2}} \end{array}$$

for approach C,

$$\begin{array}{l} {\underline{F}}_{ij}^{E\left(CD\right)}\left(r_{ij}\right)=-\left(\frac{dt_{C}\left(r_{ij}\right)}{dr_{ij}}t_{D}\left(r_{ij}\right)+t_{C}\left(r_{ij}\right)\frac{dt_{D}\left(r_{ij}\right)}{dr_{ij}}\right){\underline{r}}_{ij}^{0}\\ \frac{dt_{D}\left(r_{ij}\right)}{dr_{ij}}=\frac{1}{\lambda }\left(1+\frac{2r_{ij}}{\lambda }\right)\exp\left\{-\frac{2r_{ij}}{\lambda }\right\} \end{array}$$

for approach CD, and

$$\begin{array}{l} {\underline{F}}_{ij}^{E\left(CDS\right)}\left(r_{ij}\right)=-\left(\frac{dt_{C}\left(r_{ij}\right)}{dr_{ij}}t_{D}\left(r_{ij}\right)t_{S}\left(r_{ij}\right)+t_{C}\left(r_{ij}\right)\frac{dt_{D}\left(r_{ij}\right)}{dr_{ij}}t_{S}\left(r_{ij}\right)+t_{C}\left(r_{ij}\right)t_{D}\left(r_{ij}\right)\frac{dt_{S}\left(r_{ij}\right)}{dr_{ij}}\right){\underline{r}}_{ij}^{0}\\ \frac{dt_{S}\left(r_{ij}\right)}{dr_{ij}}=\frac{1}{R_{cutoff,el}}\left(\frac{35}{2}{\left(\frac{r_{ij}}{R_{cutoff,el}}\right)}^{6}-42{\left(\frac{r_{ij}}{R_{cutoff,el}}\right)}^{5}+\frac{105}{4}{\left(\frac{r_{ij}}{R_{cutoff,el}}\right)}^{4}-\frac{7}{4}\right) \end{array}$$

for approach CDS.

For C alone, the resulting electrostatic forces are truncated to a maximum value to attenuate the hard potential by preventing its striving to infinity (where the maximum value is set to 25 reduced DPD units, similar to the repulsion of equal particles at room temperature with an electrostatic coupling constant of 1 as evaluated in [10] and used in [11]). All electrostatic interactions are confined to an electrostatic cut-off radius of 5 or 10 reduced DPD length units.

For studying membrane disruption by lipid extraction with molecular fragment DPD (with a single water molecule being represented by a single DPD particle), the versatile “sandwich” interaction model described in [11] is used where two plasma membranes surround an enclosed cyclotide/water compartment (compare Figure 3). The model itself is an artificial construct to estimate lipid extraction using a rapid simulation technique. Due to the lack of experimental values, the choice of cyclotide number in the cyclotide/water compartment is determined by maximizing the disruptive effect at the minimum cyclotide number, where the extremes would be a single cyclotide within the compartment (with possible membrane interaction but no disruptive effect) and a biologically unrealistic compartment consisting only of cyclotides without water. The model comprises the complete partitioning of the phospholipid molecules into molecular fragment particles, as well as a particle-based spatial 3D construction of the peptides with all DPD particle–particle repulsions where molecular particle–particle connectivity is controlled by additional harmonic springs. The rates of membrane disruption are determined as outlined in [11] with a minor difference: the evaluated rates describe the percentage of outer leaflet phospholipid molecules per microsecond that was extracted from the surrounding membranes as a more evident quantity in comparison to the percentage of ethane (Et) particles in [11]. Four cyclotide/membrane systems are analyzed that span the range of detected membrane disruption activities (see details in [11]): kB1-W19Y-P20S-V21T-L27T-P28S-V29T/NoC-PM with vanishing activity over kB1-W19Y-P20S-V21T/NoC-PM and kB1/NoC-PM to cO2-E6Q/NoC-PM with the highest activity. The acronyms denote the Möbius cyclotide Kalata B1 (kB1) and the Bracelet cyclotide Cycloviolacin O2 (cO2) with possible mutations, e.g., W19Y in kB1-W19Y-P20S-V21T denotes an exchange of hydrophobic amino acid tryptophan (W) in position 19 of wild-type Kalata B1 with the more polar amino acid tyrosine (Y). NoC-PM defines a biological plasma membrane that consists of a phospholipid composition of 40% DMPC, 20% DOPE, 5% PIP_2_, 10% DOPS, and 25% SM, with an inner to outer leaflet distribution of 24 to 76 for DMPC, 77.5 to 22.5 for DOPE, 75 to 25 for PIP_2_, 1 to 0 for DOPS (only inside), and 22 to 78 for SM, but without (uncharged) cholesterol. The chosen area per lipid leads to realistic membrane thicknesses and lipid distributions that are consistent with experimental findings and alternative simulation results. The simulation box size was doubled in the x- and y-direction (i.e., quadrupled in total) compared with the simulation boxes studied in [11], which led to an x- and y-dimension of 77 reduced DPD length units (corresponding to a physical length of about 377 Å) and a z-dimension of 104 reduced DPD length units (corresponding to a physical length of about 509 Å). Periodic boundary conditions are turned on in the x- and y-direction.

All simulations are carried out with the open DPD environment MFsim [28,29], which utilizes the open Jdpd simulation kernel [3,30]. The sketched electrostatic interactions are implemented in Jdpd classes *ParticlePairElectrostaticsDpdPotentialCalculator* (for the potential energy between two charged particles) and *ParticlePairElectrostaticsDpdForceConservativeCalculator* (for the electrostatic forces of charged particles) in method *calculateParticlePairInteraction*, in which the methods could be easily extended to alternative calculation schemes (e.g., [31]). The MFsim graphical user interface is extended accordingly to allow for a comfortable setting of the electrostatic interaction parameters. All simulation job definitions are openly documented at [32] and may be viewed in full detail using the MFsim system. A simulation run of the cyclotide/membrane systems with nearly 2 million particles for 100,000 integration steps (corresponding to about 6 microseconds) performs within 15 h with 16 parallelized calculation threads. The calculation of the electrostatic interactions for charged particles requires less than 10% of the total simulation time with an electrostatic cut-off radius of 5 reduced DPD length units.

## 3. Results

**Figure 1:**
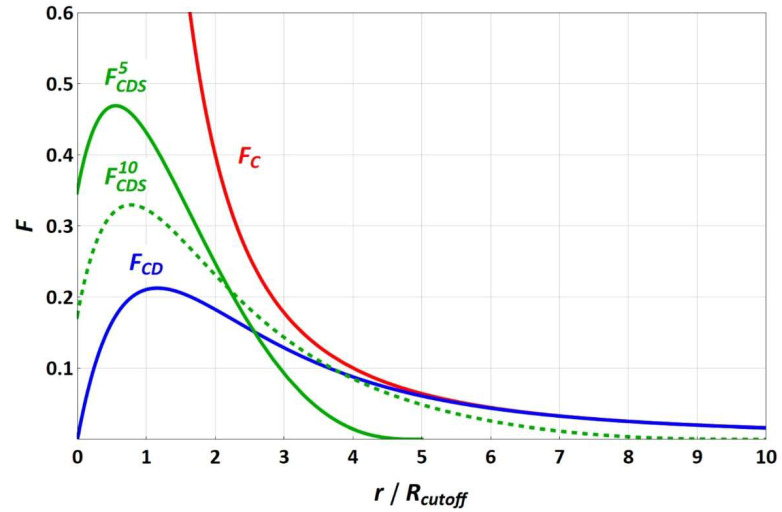
Electrostatics forces *F* (in reduced DPD units) between equally charged particles (with a single elementary charge) for the different approaches (C, CD, CDS) along a distance relative to the DPD cut-off radius *R_cutoff_* of one reduced DPD unit. Abbreviations are outlined in the text; the superscripts refer to the electrostatic cut-off radii of 5 or 10 reduced DPD length units.

The force functions of equally charged particles (with a valence of one) for the different electrostatic approaches are sketched in Figure 1. Since the splitting term depends on the electrostatic cut-off radius, the *F_CDS_* force functions are shown for the electrostatic cut-off radii of 5 and 10 reduced DPD length units, in which increasing the electrostatic cut-off radius leads to convergence of *F_CDS_* and *F_CD_*, with the splitting term approaching 1. While *F_C_* strives toward infinity for a vanishing particle–particle distance, *F_CD_* and *F_CDS_* run through a maximum toward a fixed force value that becomes zero for *F_CD_*.

**Figure 2:**
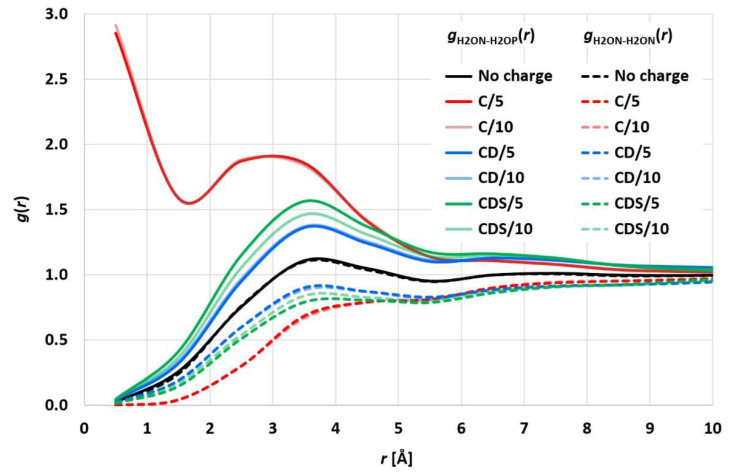
Particle–particle radial distribution functions of the pure water system with H2ON-H2OP or H2ON-H2ON ion pairs for the different approaches (C, CD, CDS) and different electrostatic cut-off radii (5 or 10 reduced DPD length units).

In [10,11], a pure Coulomb approach was used to account for electrostatics particle–particle interactions with a truncated maximum force value and the electrostatic coupling constant being empirically evaluated to avoid artificial ion pairing. As a measure of the latter, the distance between differently charged ion pairs, which were initially located at the same position, was determined throughout the simulation (see Figure 1 in [10]). The evaluated electrostatic coupling constant led to distances of the differently charged ion pairs being equal to the corresponding uncharged particle pairs. While this approach may be plausible overall and prevents the initial ion pairs from simply sticking together, it neglects possible statistically strong ion pairing beyond enrichment or depletion of the charged particle coordination shells. To analyze the statistical surroundings of charged (valence 1) particles, a simulation run of 2 million particles (denoted as water H2O particles) with 50,000 positively charged (H2OP) and negatively charged (H2ON) ion pairs (H2OP-H2ON) was performed for 40,000 integration steps (corresponding to 2 microseconds). Figure 2 shows the resulting H2ON-H2OP and H2ON-H2ON radial distribution functions (RDF), which exhibit a distinct statistical ion pairing for the pure Coulomb approach in [10,11], whereas “charge smearing” by a charge distribution (*F_CD_* or *F_CDS_*) leads to the expected statistical enrichment/depletion of the counter ion in the surrounding coordination shells. The RDFs for CD and CDS satisfy the relation *g*_H2ON-H2OP_(*r*) × *g*_H2ON-H2ON_(*r*) = *g*^2^(*r*), with *g*(*r*) being the RDF of uncharged H2O particles, which is consistent with the results of [4,25]. The different electrostatics cut-off radii of 5 and 10 reduced DPD length units lead to comparable results so that the shorter 5 reduced DPD length units (corresponding to a physical length of 22 Å) seem to be sufficient, which in turn leads to considerably decreased simulation times.

**Figure 3:**
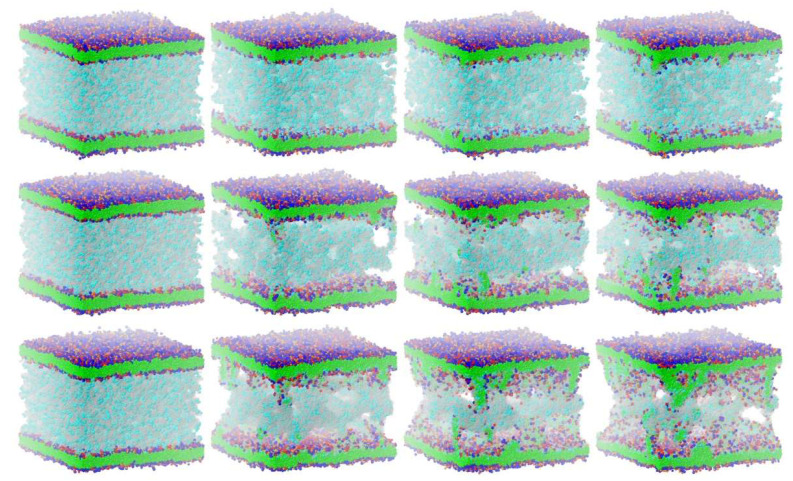
Cyclotide-induced membrane disruption by lipid extraction: Simulation box views of the “sandwich” cyclotide/membrane systems kB1-W19Y-P20S-V21T-L27T-P28S-V29T/NoC-PM (**top** row), kB1-W19Y-P20S-V21T/NoC-PM (**middle** row), and kB1/NoC-PM (**bottom** row) at 500 (first column), 35,000 (second column), 70,000 (third column), and 100,000 (fourth column) integration time steps (corresponding to 6 microseconds). Phospholipid particles in blue and red are charged, uncharged hydrophobic phospholipid particles are colored in green, cyclotide backbone particles are transparently shown in gray, with backbone particles of the cyclotides hydrophobic patch being colored in cyan. All water particles are omitted from the display, and the simulation boxes are truncated accordingly. The simulations show the CDS approach with an electrostatic cut-off radius of 5 reduced DPD length units.

The cyclotide-induced membrane disruption by lipid extraction may be qualitatively characterized as follows [11]: From their initial random start distribution, the cyclotides begin to aggregate and form oligomers that eventually develop into tubular molecular superstructures. This collaborative cyclotide network allows membrane lipids to leave their membrane environment and distribute into the cyclotide/water compartment in between the plasma membranes (see Figure 3). In the course of time, the lipids of the outer membrane leaflets (directed toward the cyclotide/water compartment) are increasingly “consumed” by cyclotide superstructures, with lipid extraction becoming more and more “saturated.” Membrane curvature or even rupture makes the quantitative evaluation procedure increasingly obsolete. Therefore, the evaluation procedure is limited to an intermediate “linear range” on the order of a few microseconds of the inherently nonlinear membrane disruption process.

The details and the extent of the membrane disruption process are determined by the individual cyclotide and membrane types. The kB1-W19Y-P20S-V21T-L27T-P28S-V29T mutant, in which the important hydrophobic patch amino acids have been completely converted to more hydrophilic alternatives, still shows cyclotide oligomerization but does no longer form tubular superstructures that support membrane disruption. Thus, this mutant exhibits negligible membrane disruption activity. In contrast, the kB1 wild-type with an intact hydrophobic patch shows significant lipid extraction, while the kB1-W19Y-P20S-V21T mutant (half of the amino acids of the hydrophobic patch have been replaced by more hydrophilic ones) is in between (Figure 3). These qualitative findings are not affected by the different electrostatic approaches, i.e., the apparent order of activity is not changed.

**Figure 4:**
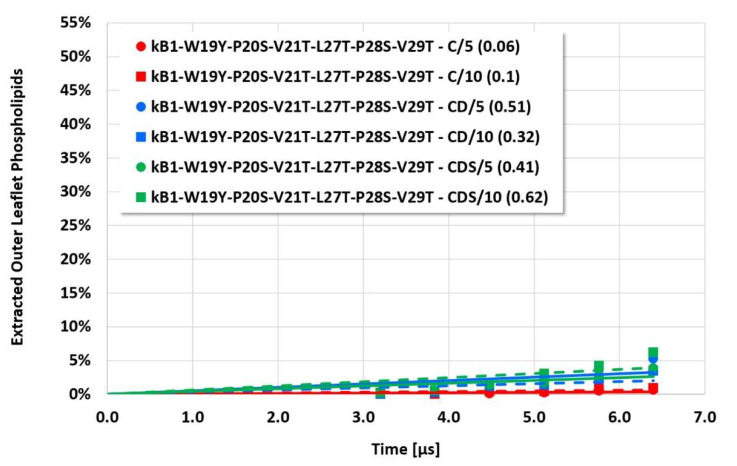
Cyclotide/membrane system kB1-W19Y-P20S-V21T-L27T-P28S-V29T/NoC-PM: Quantitative evaluation of the cyclotide-induced extraction of membrane lipids into the cyclotide/water compartment for the different approaches (C, CD, CDS) and different electrostatic cut-off radii (5 or 10 reduced DPD length units). The resulting rates of membrane disruption (percent of outer leaflet phospholipids being extracted per microsecond) are provided in brackets.

**Figure 5:**
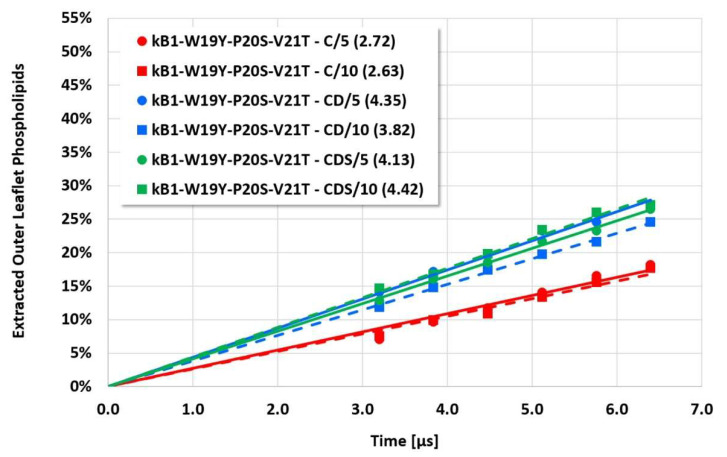
Cyclotide/membrane system kB1-W19Y-P20S-V21T/NoC-PM (see Figure 4 for details).

**Figure 6:**
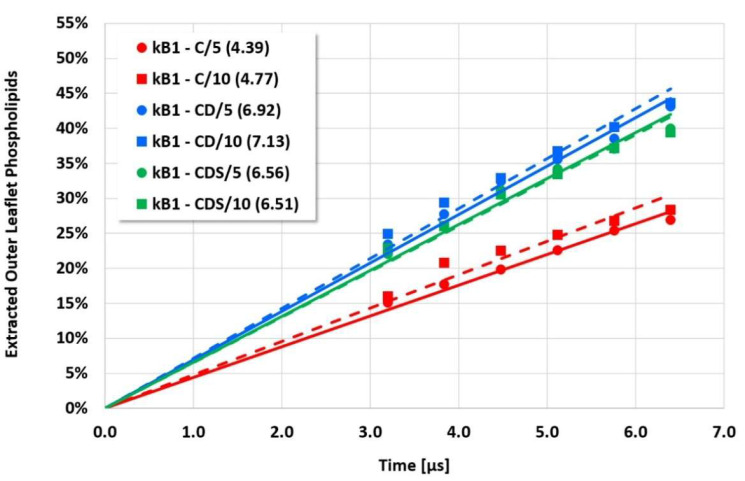
Cyclotide/membrane system kB1/NoC-PM (see Figure 4 for details).

**Figure 7:**
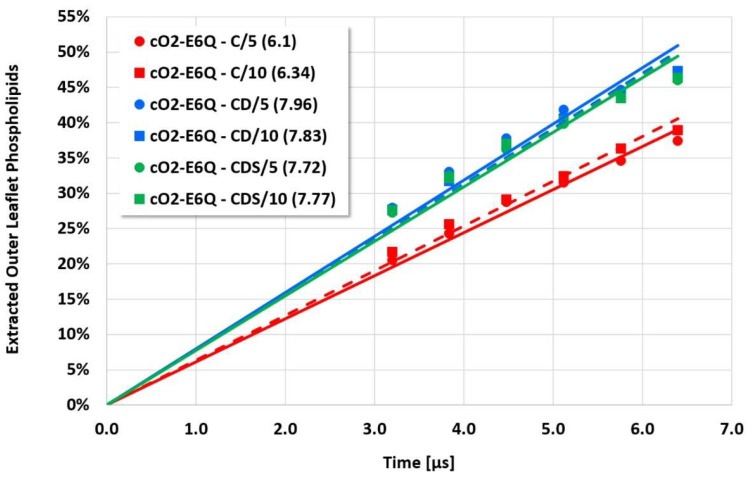
Cyclotide/membrane system cO2-E6Q/NoC-PM (see Figure 4 for details).

However, the more sensitive quantitative evaluation of lipid extraction exhibits significant differences (see Figure 4, Figure 5, Figure 6 and Figure 7). The pure electrostatic C approach leads to systematically reduced membrane disruption rates for all four cyclotide/membrane systems studied, whereas the CD and CDS approaches lead to comparable rates. A doubling of the electrostatic cut-off radius from 5 to 10 reduced DPD length units does not affect these findings. The pure C approach seems to overemphasize electrostatic interactions that lead to a specific membrane stabilization against disruptive attacks due to the charged phospholipid particles. This stabilization effect becomes more pronounced with increasing cyclotide activity for the kB1 variants, which all have the same zero net charge state (at pH 7.4). For the cO2-E6Q mutant, the stabilization effect is less pronounced in comparison to kB1, although its activity is higher. This may be traced to its different net charge state (+3 at pH 7.4), whereby the pure C approach may lead to an increased activity for this highly charged cyclotide that counteracts the membrane stabilization.

## 4. Discussion

Complex simulation models require numerous settings that follow theoretical considerations, deliberate choices, or simply experience. The influence of specific settings may vary for different areas of application. From a chemical point of view, the inclusion of electrostatic interactions is mandatory for an adequate description of biological membranes or biomolecules such as peptides or proteins.

In a DPD framework, a simple Coulomb approach with truncated forces tuned to avoid individual strong ion pairing still reveals hidden statistical pairing effects that may lead to artificial stabilization of molecular superstructures such as membranes or distortion of cyclotide activity depending on the cyclotide’s charge state. While qualitative behavior is not affected in an apparent manner, more sensitive quantitative evaluations can be systematically biased. This was demonstrated in the complex disruptive interaction of cyclotides with a biological plasma membrane. The findings suggest a charge smearing of point charges by an adequate charge distribution. Since mesoscopic simulation boxes are large, there is only a negligible influence of mirror charges due to periodic boundary conditions. On the other hand, the comparable results of the CD and CDS approaches, in combination with their insensitivity to different electrostatics cut-off radii, show that it is advised to resist the temptation to overstretch the discussion of minor differences in model setups on the mesoscale. Adequate handling of charges may be particularly useful in studying the influence of a cyclotide’s charge state on its membrane-disrupting activity, which is currently not well understood.

## Data Availability

All simulation job definitions are openly documented at [32]. The open MFsim simulation environment with the open Jdpd simulation kernel is available at [29].

## References

[1] Kutzner C., Páll S., Fechner M., Esztermann A., de Groot B.L., Grubmüller H. (2019). More Bang for Your Buck: Improved use of GPU Nodes for GROMACS 2018. J. Comput. Chem..

[2] Espanol P., Warren P.B. (2017). Perspective: Dissipative particle dynamics. J. Chem. Phys..

[3] Van den Broek K., Kuhn H., Zielesny A. (2018). Jdpd—An open java simulation kernel for molecular fragment dissipative particle dynamics. J. Cheminf..

[4] Groot R.D. (2003). Electrostatic interactions in dissipative particle dynamics—Simulation of polyelectrolytes and anionic surfactants. J. Chem. Phys..

[5] De Veer J., Kan M.W., Craik D.J. (2019). Cyclotides: From Structure to Function. Chem. Rev..

[6] Burman R., Strömstedt A.A., Malmsten M., Göransson U. (2011). Cyclotide–membrane interactions: Defining factors of membrane binding, depletion and disruption. Biochim. Biophys. Acta.

[7] Nawae W., Hannongbua S., Ruengjitchatchawalya M. (2014). Defining the Membrane Disruption Mechanism of Kalata B1 via Coarse-grained Molecular Dynamics Simulations. Sci. Rep..

[8] Nawae W., Hannongbua S., Ruengjitchatchawalya M. (2014). Dynamic Scenario of Membrane Binding Process of Kalata B1. PLoS ONE.

[9] Nawae W., Hannongbua S., Ruengjitchatchawalya M. (2017). Molecular dynamics exploration of poration and leaking caused by kalata B1 in HIV-infected cell membrane compared to host and HIV membranes. Sci. Rep..

[10] Truszkowski A., van den Broek K., Kuhn H., Zielesny A., Epple M. (2015). Mesoscopic Simulation of Phospholipid Membranes, Peptides, and Proteins with Molecular Fragment Dynamics. J. Chem. Inf. Model..

[11] Van den Broek K., Epple M., Kersten L.S., Kuhn H., Zielesny A. (2021). Quantitative Estimation of Cyclotide-Induced Bilayer Membrane Disruption by Lipid Extraction with Mesoscopic Simulation. J. Chem. Inf. Model..

[12] Hoogerbrugge P.J., Koelman J.M.V.A. (1992). Simulating Microscopic Hydrodynamic Phenomena with Dissipative Particle Dynamics. Europhys. Lett..

[13] Koelman J.M.V.A., Hoogerbrugge P.J. (1993). Dynamic Simulations of Hard-Sphere Suspensions Under Steady Shear. Europhys. Lett..

[14] Espanol P., Warren P. (1995). Statistical Mechanics of Dissipative Particle Dynamics. Europhys. Lett..

[15] Espanol P. (1995). Hydrodynamics from dissipative particle dynamics. Phys. Rev. E.

[16] Groot R.D., Warren P. (1997). Dissipative particle dynamics: Bridging the gap between atomistic and mesoscopic simulation. J. Chem. Phys..

[17] Groot R.D., Madden T.J. (1998). Dynamic simulation of diblock copolymer microphase separation. J. Chem. Phys..

[18] Ryjkina E., Kuhn H., Rehage H., Müller F., Peggau J. (2002). Molecular Dynamic Computer Simulations of Phase Behavior of Non-Ionic Surfactants. Angew. Chem. Int. Ed..

[19] Schulz S.G., Kuhn H., Schmid G., Mund C., Venzmer J. (2004). Phase behavior of amphiphilic polymers: A dissipative particles dynamics study. Colloid Polym. Sci..

[20] Truszkowski A., Epple M., Fiethen A., Zielesny A., Kuhn H. (2013). Molecular fragment dynamics study on the water–air interface behavior of non-ionic polyoxyethylene alkyl ether surfactants. J. Colloid Interface Sci..

[21] Vishnyakov A., Lee M.-T., Neimark A.V. (2013). Prediction of the Critical Micelle Concentration of Nonionic Surfactants by Dissipative Particle Dynamics Simulations. J. Phys. Chem. Lett..

[22] Truszkowski A., Daniel M., Kuhn H., Neumann S., Steinbeck C., Zielesny A., Epple M. (2014). A molecular fragment cheminformatics roadmap for mesoscopic simulation. J. Cheminf..

[23] Wolf D., Keblinski P., Phillpot S.R., Eggebrecht J. (1999). Exact method for the simulation of Coulombic systems by spherically truncated, pairwise r^−1^ summation. J. Chem. Phys..

[24] Fennell C.J., Gezelter J.D. (2006). Is the Ewald summation still necessary? Pairwise alternatives to the accepted standard for long-range electrostatics. J. Chem. Phys..

[25] González-Melchor M., Mayoral E., Velázquez M.E., Alejandre J. (2006). Electrostatic interactions in dissipative particle dynamics using the Ewald sums. J. Chem. Phys..

[26] Fanourgakis G.S. (2015). An Extension of Wolf’s Method for the Treatment of Electrostatic Interactions: Application to Liquid Water and Aqueous Solutions. J. Phys. Chem. B.

[27] Malmberg C.G., Maryott A.A. (1956). Dielectric Constant of Water from 0 to 1000 C. J. Res. Natl. Bur. Stand..

[28] Van den Broek K., Daniel M., Epple M., Hein J.M., Kuhn H., Neumann S., Truszkowski A., Zielesny A. (2020). MFsim—An open Java all-in-one rich-client simulation environment for mesoscopic simulation. J. Cheminf..

[29] MFsim—An Open Java All-in-One Rich-Client Simulation Environment for Mesoscopic Simulation. https://github.com/zielesny/MFsim.

[30] Jdpd—An Open Java Simulation Kernel for Molecular Fragment Dissipative Particle Dynamics (DPD). https://github.com/zielesny/Jdpd.

[31] Gavrilov A.A., Chertovich A.V., Kramarenko E.Y. (2016). Dissipative particle dynamics for systems with high density of charges: Implementation of electrostatic interactions. J. Chem. Phys..

[32] 2022 Cyclotide-Membrane Electrostatics Study Subfolder in GitHub repository. https://github.com/zielesny/MFsim.

